# Sex differences in lymphoid follicles in COPD airways

**DOI:** 10.1186/s12931-020-1311-8

**Published:** 2020-02-07

**Authors:** Anthony Tam, Naoya Tanabe, Andrew Churg, Joanne L. Wright, James C. Hogg, Don D. Sin

**Affiliations:** 1 Centre for Heart Lung Innovation, St. Paul’s Hospital, & Department of Medicine, Vancouver, British Columbia Canada; 2 Department of Respiratory Medicine, Graduate School of Medicine, Kyoto University, Kyoto, Japan; 3 Department of Pathology, University of British Columbia, Vancouver, British Columbia Canada

**Keywords:** Sex differences, Oxidative stress, Lymphoid follicles

## Abstract

**Background:**

Female smokers have increased risk for chronic obstructive pulmonary disease (COPD) compared with male smokers who have a similar history of cigarette smoke exposure. Tertiary lymphoid follicles are often found in the lungs of patients with severe COPD but sex-related differences have not been previously investigated. We determined the impact of female sex hormones on chronic cigarette smoke-induced expression of lymphoid aggregates in mice with COPD-like pathologies.

**Methods:**

Lymphoid aggregate counts, total aggregate cross-sectional area and foamy macrophage counts were determined morphometrically in male, female, and ovariectomized mice exposed to air or cigarette smoke for 6 months. B-cell activating factor (BAFF) protein expression and markers of oxidative stress were evaluated in mouse lung tissues by immunofluorescence staining and gene expression analyses. Quantitative histology was performed on lung tissue sections of human COPD lungs to evaluate follicle formation.

**Results:**

Lymphoid follicle and foamy macrophage counts as well as the total follicle cross-sectional area were differentially increased in lung tissues of female mice compared to male mice, and these differences were abolished by ovariectomy. These lymphoid aggregates were positive for CD45, CD20, CD21 and BAFF expression. Differential increases in *Mmp12 and Cxcl2* gene expression correlated with an increase in foamy macrophages in parenchymal tissues of female but not male mice after smoke exposure. Parenchymal tissues from female mice failed to induce antioxidant-related genes in response to smoke exposure, and this effect was restored by ovariectomy. 3-nitrotyrosine, a stable marker of oxidative stress, positively correlated with *Mmp12* and *Cxcl2* gene expression. Hydrogen peroxide induced BAFF protein in mouse macrophage cell line. In human lung tissues, female smokers with severe COPD demonstrated increased numbers of lymphoid follicles compared with males.

**Conclusions:**

Chronic smoke exposure increases the risk of lymphoid aggregate formation in female mice compared with male mice, which is mediated female sex hormones and BAFF expression in an oxidative environment.

## Background

Chronic obstructive pulmonary disease (COPD) is the third most prevalent cause of mortality in North America and Western Europe, and imposes a tremendous economic burden to the healthcare system [1]. Although both men and women develop COPD, females are at a greater risk of developing significant COPD with lower exposures to tobacco smoke. For example, female smokers are three times more likely to develop severe COPD (defined as lung function less than 50% of predicted before 55 years of age) compared to male smokers with the same smoking history [2–4]. We have also shown in a murine model that females exposed to 6 months of tobacco smoke develop a greater burden of COPD-like changes in the airways compared with males, but these differences disappeared when female mice were ovariectomized and then exposed to cigarette smoke [5]. The mechanism(s) for sexual dimorphism in the risk of early emphysema vs airway disease in patients with COPD are unknown. One plausible mechanism is dysregulation in the local immune response to antigens. We have shown previously that there is a 6 to 7 fold increase in the percentage of small airways containing lymphoid follicles among patients with COPD (compared with smokers without COPD) [6]. These lymphoid follicles contain B-cells, follicular dendritic cells and T-cells [7]. Consistent with these clinical observations, mice chronically exposed to cigarette smoke (which leads to COPD-like changes in the lung) also harbor lymphoid aggregates [8–11]. However, B cell-deficient mice are completely protected from chronic smoke-induced lymphoid aggregate formation and do not develop emphysema [8]. These aggregates are often surrounded by foamy macrophages that are thought to contain cigarette smoke-induced lipid oxidation products [11] and associate with chronic inflammatory processes [12]. The role of sex and specifically female sex hormones in the pathogenesis of lymphoid follicles in small airways has not been previously investigated. Our primary hypothesis was that females would have increased expression of lymphoid follicles in the airways of COPD lungs and that ovariectomy would prevent the formation of these lymphoid aggregates in mice.

## Methods

### Animals

As described previously [13], adult male, female and ovariectomized C57BL/6 mice (12 weeks old) were obtained from Charles River (Montreal, PQ, Canada). Surgical ovariectomy of female mice was performed at Charles River 4 weeks prior to cigarette smoke exposure. 1R1 and 2R4F research grade cigarettes were obtained from the University of Kentucky (Lexington, KY). All animal procedures were approved by the University of British Columbia Animal Care Committee (A11–0149).

### Smoke exposure

As described previously [13], we studied 6 groups of mice: 1) *N* = 10 male controls, 2) *N* = 12 male smoke-exposed, 3) *N* = 10 female controls, 4) *N* = 12 female smoked-exposed, 5) N = 10 ovariectomized controls, and 6) N = 12 ovariectomized smoke-exposed. The smoke-exposed groups were exposed to three cigarettes (one 1R1 and two 2R4F with the filters removed, or two 1R1 and one 2R4F with the filters removed on every other smoking day) per day for 5 days per week for 6 months. All smoke exposures were conducted using our standard nose-only smoke exposure system [5, 14, 15].

### Histology

The lungs were inflated at 25cmH_2_0 and fixed under pressure in formalin for 24 h. Formalin-fixed paraffin-embedded (FFPE) sagittal sections (5 μm) were stained with Hematoxylin and Eosin (H&E) for histopathological assessment of lymphoid aggregates and foamy macrophages. High-resolution images were generated using a ScanScope Slide Scanner (Aperio Technologies, Vista, CA, USA). The number of vessel-associated, bronchial-associated and parenchymal-associated lymphoid aggregates containing > 50 cells in a dense cluster were quantified and normalized to the total lung cross-sectional area in cm^2^. Fractional area of lymphoid aggregates and foamy macrophage counts were quantified and normalized per total lung cross-sectional area.

### Immunohistochemical staining

FFPE mouse and/or human lung tissues were stained with antibodies against CD79α-B cell marker (M7050, Dako), B-cell activating factor-BAFF (Ab117256, Abcam) and CD21-follicular dendritic cell marker (Ab75985, Abcam) and Ki67-proliferation marker (Ab16667, Abcam) using the Bond Polymer Refine Red Detection kit on the Leica Bond Autostainer according to the manufacturer’s protocol as previously described [16]. Slides were scanned using the Aperio imaging system (Leica Biosystem; Concord, Ontario).

### Immunofluorescence staining for confocal microscopy

FFPE mouse lung tissue sections were dewaxed, antigen retrieved at pH 6 and incubated with primary antibodies against CD45-pan leukocyte marker (Ab10558, Abcam), CD20-B cell marker (Ab64088, Abcam), BAFF (Ab117256, Abcam) and macrophages (MAB1407P, Millipore) overnight at 4 °C and stained with Alexa Fluor® 488 goat anti-mouse IgG and 594 goat anti-rabbit IgG (Life Technologies) for 2 h at room temperature. Slides were counterstained with DAPI (10,236,276,001, Sigma), cover-slipped and visualized using confocal microscopy.

### Laser-capture micro-dissection, real time PCR and oil red staining

Whole left lungs were inflated with 50% optimal cutting temperature (OCT) compound for frozen tissues in DEPC-treated water and snap frozen. Frozen lungs were cut into 5 μm-thick sections using a cryostat (Leica Biosystems, Concord, ONT) and laser capture microdissection was performed using the Leica LMD6500 Laser Microdissection System (Leica Microsystems, Concord, ONT). Parenchymal tissues were collected separately in an RLT buffer-containing Eppendorf 500-μl tube (Mississauga, ON, Canada) and stored at -80 °C until the RNA extraction procedure. Total RNA (50 ng) was reverse transcribed into cDNA using the iScript cDNA synthesis kit according to the manufacturer’s protocol (Biorad, Mississauga, ON). Real time PCR was performed on an Applied Biosystems platform (Applied Biosystems-ABI, Carlsbad, CA). Gene expression quantification was based on the ΔCT method of relative quantification with normalization to beta-2 macroglobulin (β2m); 5 μm-thick OCT-inflated frozen left lung sections were stained with oil red to visualize lipid-filled cellular contents. High-resolution images were generated using a ScanScope Slide Scanner (Aperio Technologies, Vista, CA, USA).

### Cell culture

Mouse macrophage cell line (RAW 264.7; ATCC TIB-71) was cultured in DMEM (Gibco BRL; Invitrogen, Carlsbad, CA) with 10% fetal bovine serum (FBS). To determine whether oxidative stress stimulates BAFF protein expression, RAW 264.7 cells were treated with 0.5 mM hydrogen peroxide for 24 h [17]. Cells were stained with antibodies against macrophages (MAB1407P, Millipore) and BAFF (Ab117256) was visualized using confocal microscopy.

### Human study

We analyzed histological data from lungs of COPD patients and non-COPD controls that were obtained in a previous study [6] and archived in a tissue registry approved by the Providence Health Care Research Ethics Board (H011–50110), University of British Columbia. The sample set included: 29 male and 17 female smokers, who had severe or very severe COPD based on the Global Initiative for Chronic Obstructive Lung Disease (GOLD) definition [18]. The severity of COPD was categorized based on GOLD grades (Grade 1: mild, Grade 2: moderate, Grade 3, severe and Grade 4, very severe). In this study, we examined the histological data of all small airways, and quantitated the airway-associated lymphoid follicles. This was expressed as a percentage of small airways (whose entire perimeter was between 2 and 4 mm), which contained a lymphoid follicle. We excluded airways smaller than 2 mm because their walls are generally very thin and cannot hold follicles. Subject selection criteria and demographics are shown in Additional file 1: Figure S1 and Table 1, respectively.

**Table 1:** Demographic of male and female subjects for the assessment of airway-associated lymphoid follicles

	Male	Female
No. subjects	29	19
Age	65 ± 8	67 ± 5
FEV1 (% of predicted)	23 ± 6	28 ± 8
FEV1/FVC (%)	29 ± 5*	35 ± 6
Smoking pack-year	72 ± 34	57 ± 14
No. of airways examined/subjects	6 ± 4	7 ± 5

*Pbm* basement membrane length

* *P* < 0.05 compared to female

### Statistical methods

Data were tested for normality prior to the selection of a parametric (normal distribution) or Mann Whitney (non-normal distribution) t-test, Kruskal Wallis multiple comparisons test, one-way ANOVA with Bonferroni’s multiple comparisons test, and linear regression test, where appropriate. All data were analyzed using GraphPad Prism version 8 (GraphPad Software Inc., San Diego, CA, USA) and were expressed as mean ± SEM. Statistical significance was considered at *P* < 0.05.

## Results

### Female mice have more lung-associated lymphoid aggregates than male mice with chronic smoke exposure, and these differences disappeared following ovariectomy

**Fig. 1:**
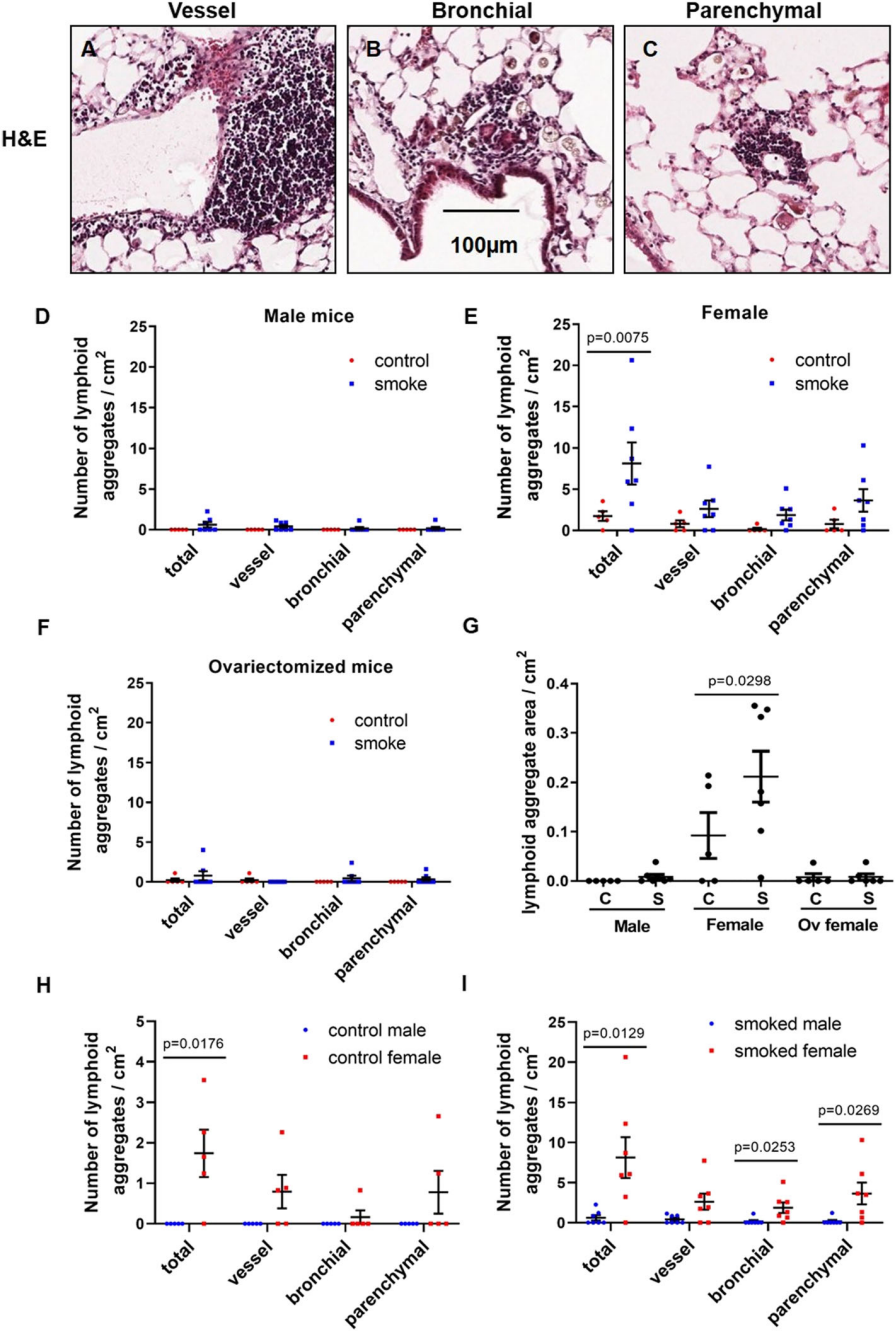
Female mice have more lymphoid follicles than male mice after chronic smoke exposure, and this effect was attenuated by ovariectomy. Representative images of **a**) vessel-associated, **b**) bronchial-associated and **c**) parenchymal-associated lymphoid aggregates in lung cross sections from smoke-exposed female mice were stained with H&E. Scale bar = 100 μm. The number of lymphoid aggregates per area (cm^2^) was quantified and compartmentalized (vessel-, bronchial- and parenchymal-associated) in lung sections of air-exposed (control) and smoke-exposed **d**) male, **e**) female and **f**) ovariectomized mice. **g**) Fractional area of lymphoid aggregates was quantified and normalized to the total lung cross-sectional area using the Aperio Imaging system. The number of lymphoid aggregates per area (cm^2^) was quantified and compartmentalized (vessel-, bronchial- and parenchymal-associated) in lung sections of H) air-exposed (control) male vs. female mice, and I) smoke-exposed male vs female mice. Values were expressed as mean ± SEM with *N* = 5–7 per group. Parametric t-test was performed in panels D-F and H-I. One-way ANOVA with Bonferroni’s multiple comparisons tests were performed in panel G.

**Fig. 2:**
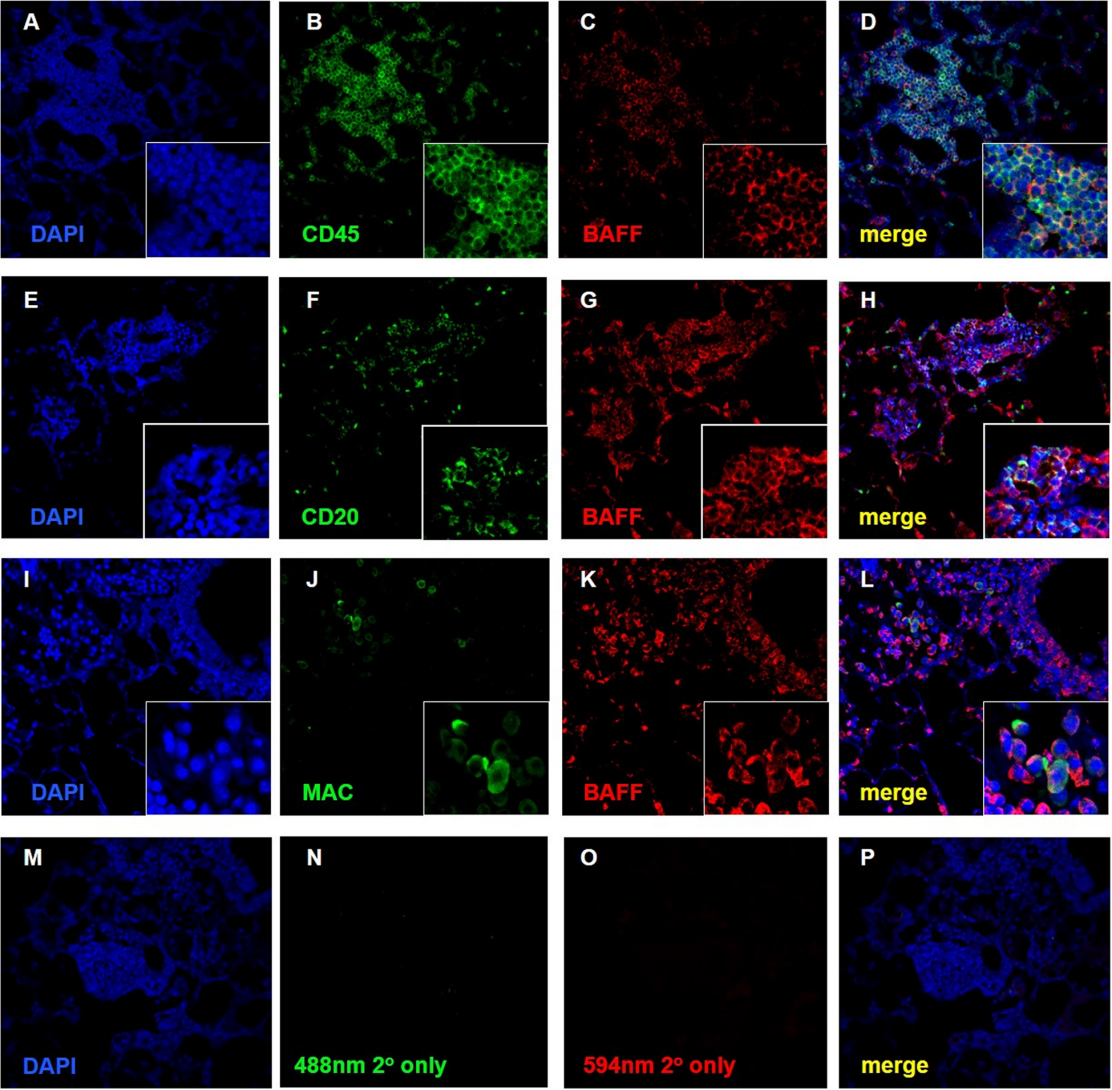
Lymphoid follicles from female smoke-exposed mice expressed CD45+ and CD20+ cells with infiltrating macrophages co-expressed with BAFF. Female smoke-exposed mouse lung sections were stained antibodies against CD45 (green) and BAFF (red) (panels **a-d**), CD20 (green) and BAFF (red) (panels **e-h**), macrophage (green) and BAFF (red) (panels **i-l**), and secondary only (panel **m-p**). Nuclei were counterstained with DAPI (blue). Green and red fluorescent staining were merged as indicator of co-localization (yellow).

To identify whether there was any sexual dimorphism in lymphoid aggregate formation in mice with COPD-like pathologies, lung tissues of male, female and ovariectomized mice exposed to air (control) or smoke for 6 months were stained with H&E for histological assessment. Representative images of vessel, bronchial- and parenchymal-associated lymphoid aggregates in the lungs of female smoke-exposed mice are shown in Fig. 1a-c. We observed a differential increase in the total number of lymphoid aggregates per cm^2^ in lung tissues of female versus male mice with chronic smoke exposure, and these differences disappeared with ovariectomy (Fig. 1d-f). Lymphoid aggregate area per lung tissue cross-sectional area was also differentially increased in female compared with male mice after smoke exposure, and this effect was abolished by ovariectomy (Fig. 1g). Female mice had greater numbers of total lymphoid aggregates than male mice at baseline (without smoke exposure) (Fig. 1h). After smoke exposure, female mice demonstrated increased numbers of bronchial, parenchymal and total lymphoid aggregates compared with male smoked mice (Fig. 1i). Representative images of lung tissues from female smoke-exposed mice revealed that these lymphoid aggregates, which were CD45+ (Fig. 2a-d), CD20+ (Fig. 2e-h) and macrophages (Fig. 2i-l), co-expressed the BAFF protein. Tissue-matched negative control lung sections were stained with secondary antibodies in Fig. 2m-p.

### Female mice have more foamy macrophages in lungs than male mice with chronic smoke exposure, and these differences were abolished with ovariectomy

**Fig. 3:**
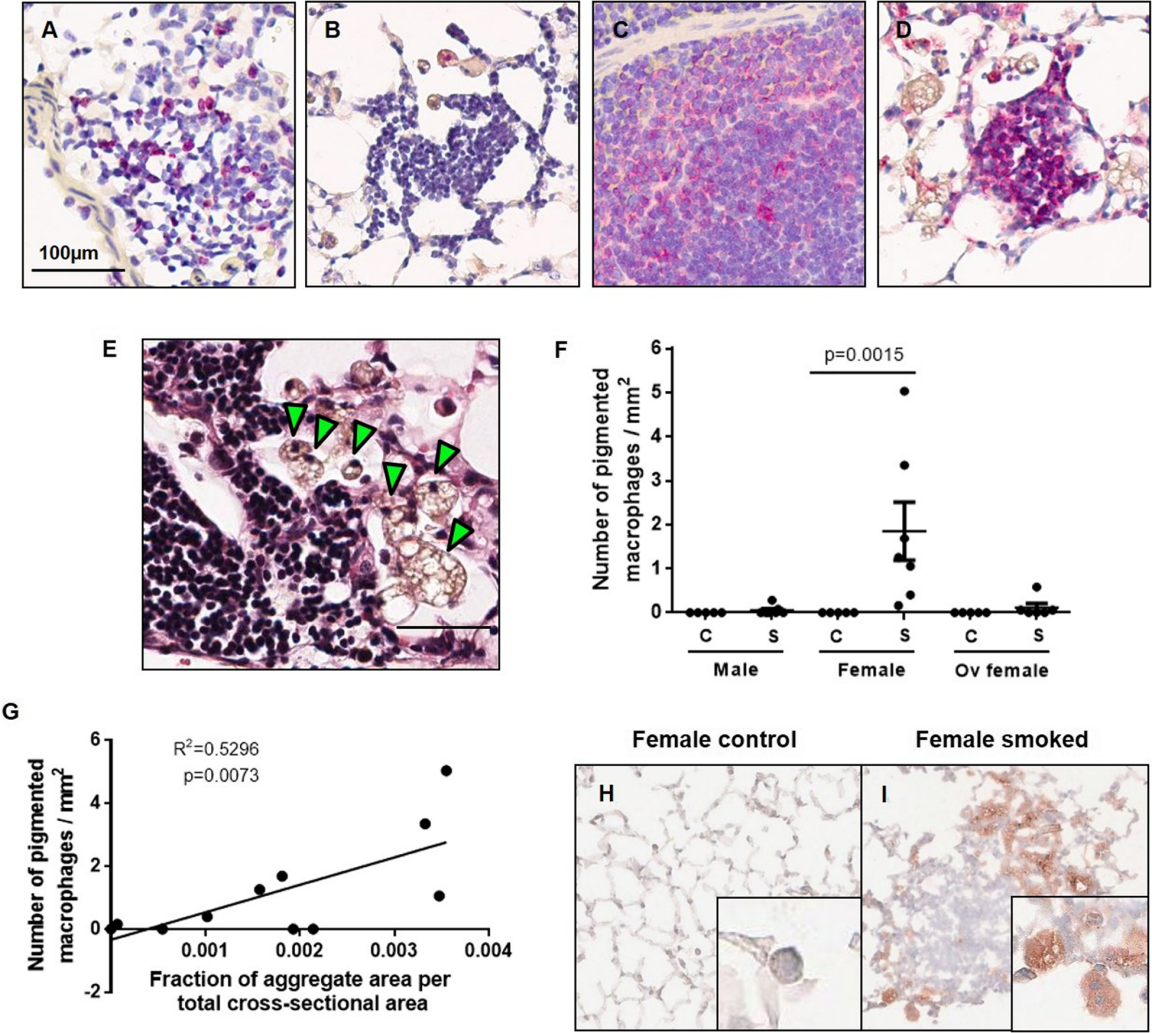
BAFF protein is differentially expressed in lung tissues of female mice with infiltration of foamy macrophages in close proximity to lymphoid follicles; Representative images stained for Ki67 (marker of proliferation) in **a**) house-dust mite exposed mouse as positive control and **b**) female smoke-exposed mouse, and CD21 (marker of follicular dendritic cells) in **c**) mouse spleen as positive control and **d**) female smoke-exposed mouse are shown. **e**) Paraffin-embedded lung section showing foamy macrophages (green arrows) located in close proximity to lymphoid follicles is stained with H&E. Scale bar = 50 μm. **f**) The number of foamy macrophages was normalized to total cross-sectional area (mm^2^). **g**) Total number of foamy macrophages per total cross-sectional area was correlated between fractions of follicle area per total cross-sectional area in female mice exposed to air or smoke for 6 months. Representative frozen lung sections from a **h**) female control and **i**) smoke-exposed female mouse stained with oil red are shown. Values were expressed as mean ± SEM with N = 5–7 per group. Panel **f** was analyzed using an one-way ANOVA with Bonferroni’s multiple comparisons test. Linear regression analysis was used in panel **g**.

Representative images showed CD21+ in all but no Ki67+ staining in all lymphoid aggregates of female smoke-exposed mice (Fig. 3a-d). Mouse spleen and house-dust mite exposed lung tissues were used as positive controls for CD21 and Ki67 staining, respectively. Consistent with previous reports [11, 12], we showed that foamy macrophages (green arrows) were located in close proximity to the lymphoid aggregates (Fig. 3e). There were significantly more foamy macrophages in whole lung tissues in female than in male or ovariectomized mice following chronic smoke exposure (Fig. 3f). In control and smoke-exposed female mice, the total number of foamy macrophages positively correlated with fractional lymphoid aggregate area of the lung (Fig. 3g). Frozen lung tissue sections showed that these foamy macrophages stained positively with oil red and were infiltrated in the peripheral regions with lymphoid aggregates; these cells were absent in control female mice (Fig. 3h-i).

### Antioxidant-related genes are differentially decreased in female mice, and 3-nitrotyrosine is positively associated with *Mmp12* and *Ccxl2* gene expression

**Fig. 4:**
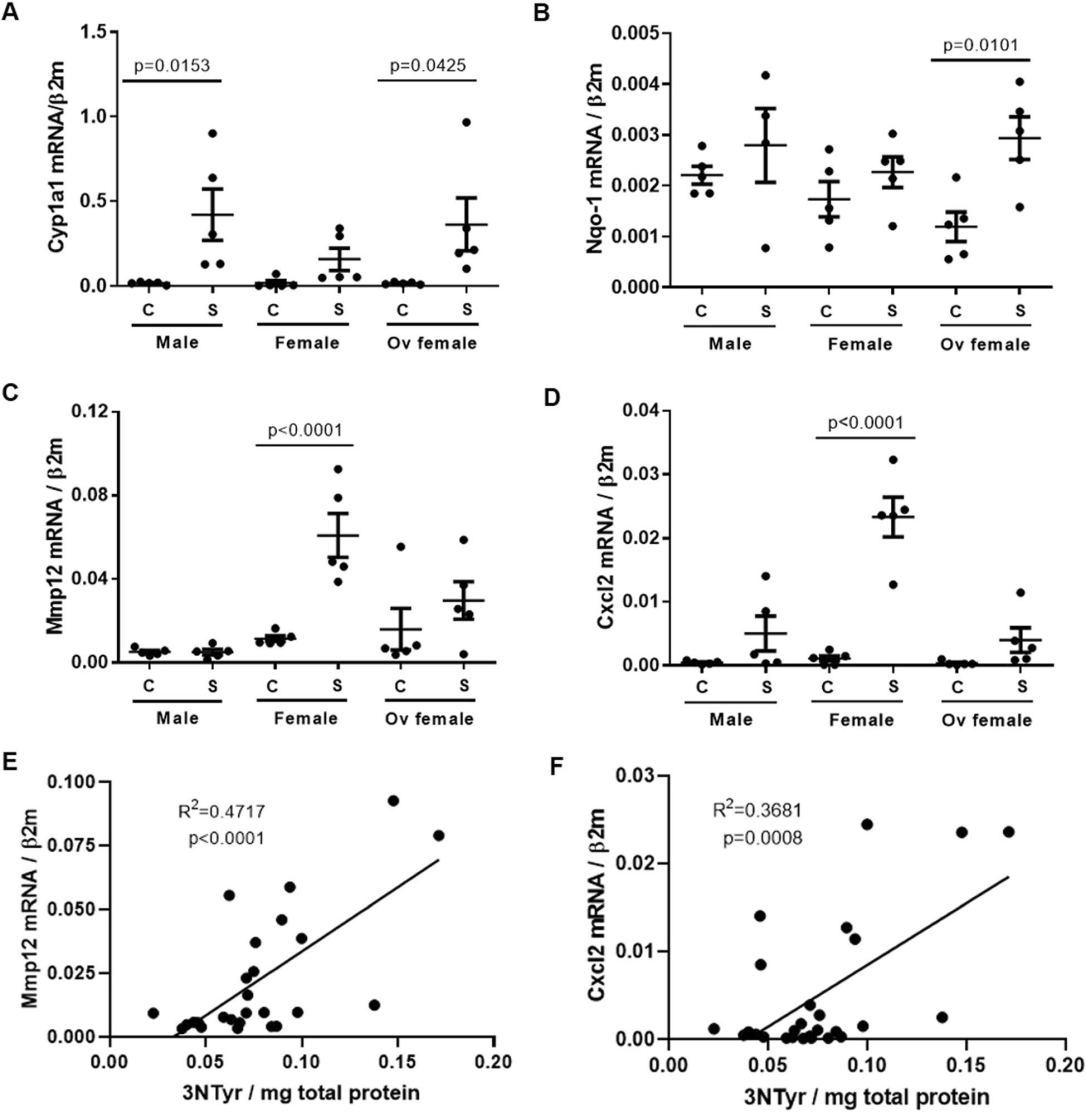
Antioxidant genes are blunted in parenchymal tissues of female mice and, *Mmp12* and *Cxcl2* genes are positively correlated with 3NTyr. **a**) *Cyp1a1* and **b**) *Nqo-1*, **c**) *Mmp12* and **d**) *Cxcl2* gene expression was normalized to *β2m* in parenchymal tissues of male, female and ovariectomized mice exposed to air (control, C) or smoke (S) for 6 months. Values were expressed as mean ± SEM (*n* = 4–5). E) *Mmp12* and F) *Cxcl2* gene expression was correlated with 3-nitrotyrosine (3NTyr) per mg of total protein. One-way analysis of variance with Bonferroni’s multiple comparisons test was used in panels **a-d**. Linear regression analyses were used panels E-F. *Cyp1a1* = cytochrome p450; *Nqo1* = NAD(P)H:quinone oxidoreductase 1, Mmp12 = matrix metalloproteinase, Cxcl2 = chemokine ligand 2 (macrophage inflammatory protein 2-alpha); *β2m* = β2-macroglobulin.

**Fig. 5:**
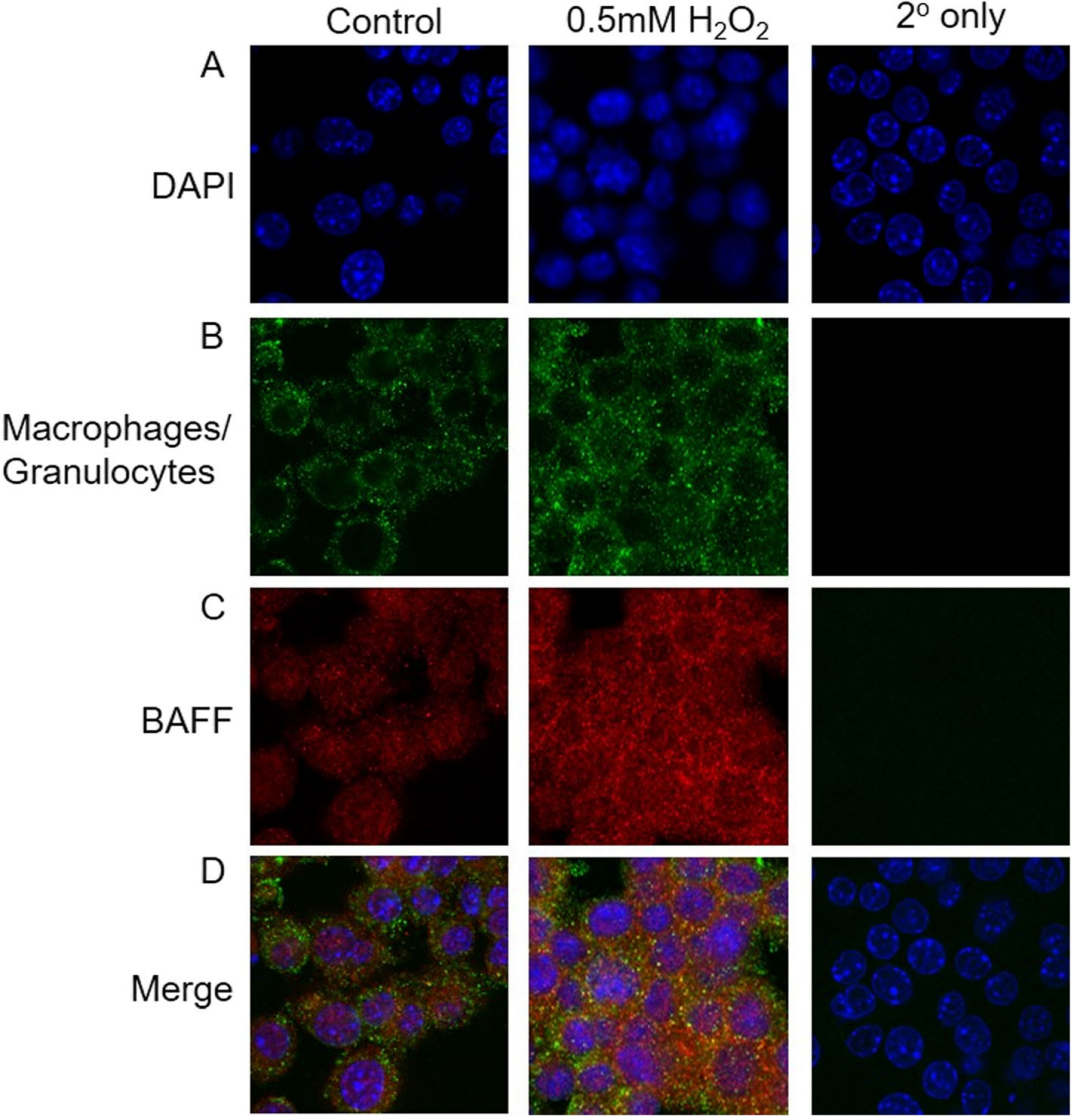
Hydrogen peroxide induces BAFF protein expression in mouse macrophage cell line. Representative immunofluorescence images of mouse macrophage cell line (RAW 264.7) stained with **a**) DAPI, **b**) macrophage/granulocytes (green) and **c**) BAFF (red) antibodies with **d**) merged images in control, 0.5 mM H_2_O_2_ and secondary antibody only-treated cells were shown.

To determine the potential molecular drivers associated with the expression of lymphoid aggregates and foamy macrophages in the lung tissues of smoke-exposed female mice, we performed expression measurements of a select number of genes on laser-captured microdissected parenchymal tissues. This showed that female mice had a blunted antioxidant response, as noted by cytochrome P450 1a1 (*Cyp1a1*) and NAD(P) H Quinone Dehydrogenase (*Nqo1*) genes, related to chronic smoke exposure, and this effect was restored by ovariectomy (Fig. 4a-b). Similarly, matrix metalloproteinase (*Mmp12*) and chemokine C-X-C motif ligand 2 (*Cxcl2*) gene expression was differentially increased in smoke-exposed female mice, which was positively associated with 3-nitrotyrosine, a stable marker of oxidative stress (Fig. 4c-f). Representative confocal images showed that hydrogen peroxide induced BAFF protein expression in mouse macrophage cell line (RAW 264.7) (Fig. 5a-d).

### Females demonstrated more airway-associated lymphoid follicles than males with severe COPD

**Fig. 6:**
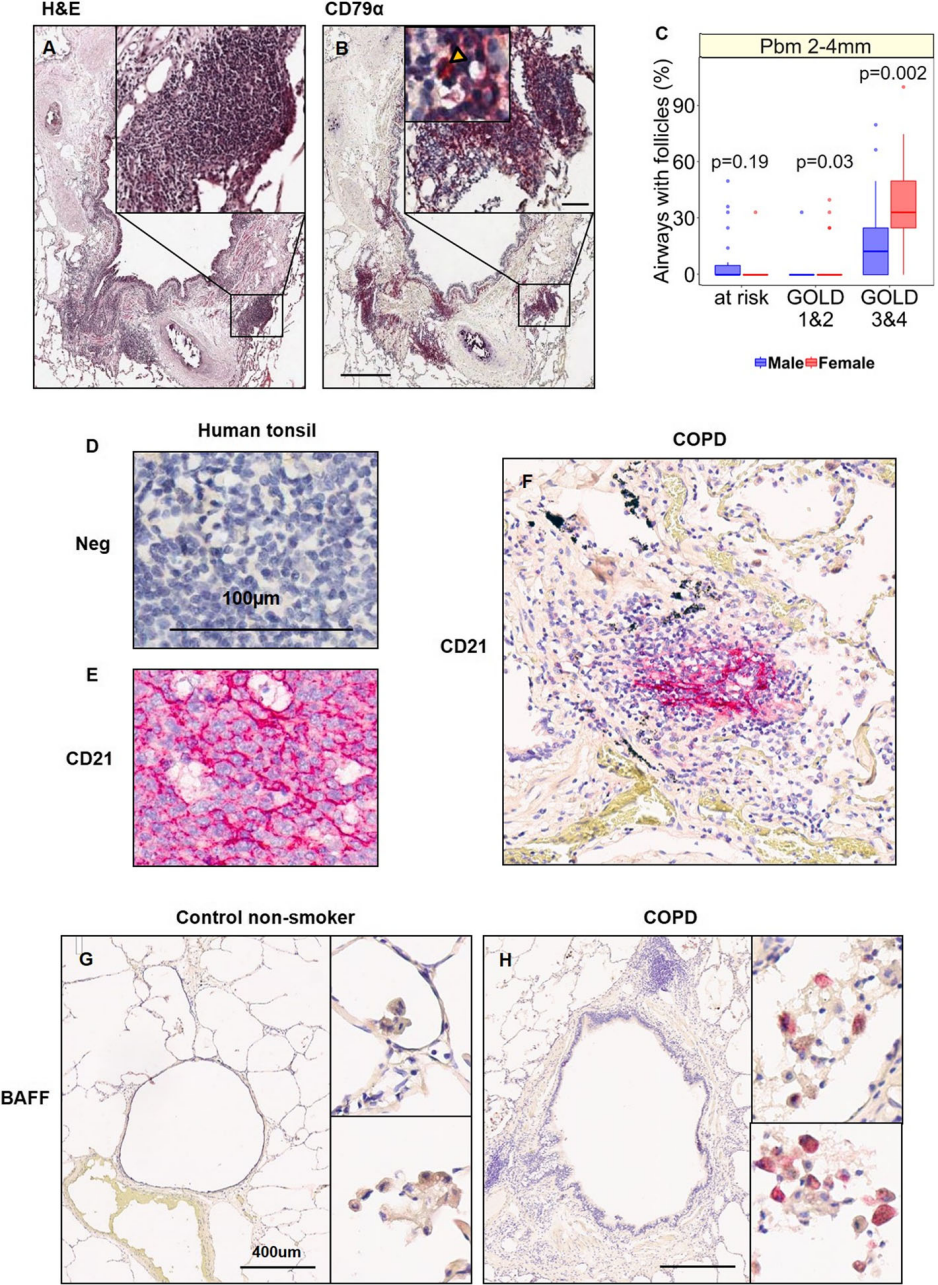
Females with severe COPD have more airway-associated lymphoid follicles in airway perimeter of 2-4 mm than those of males. Representative lung sections from a COPD patient stained with **a**) H&E and **b**) antibody against CD79α (B-cell marker; indicated by yellow triangle) in airway-associated lymphoid follicles are shown. **c** Percent airways with follicles are shown in lung tissue sections from subjects with GOLD 3–4 COPD in airways with perimeter 2-4 mm measured at the basement membrane (Pbm). Boxplots indicate the median and the interquartile range. Wilcoxon rank sum test was used in panel **c**. Human tonsil sections stained with **d**) secondary only (neg-negative control) and **e**) antibody against CD21 (follicular dendritic cell marker) were used as positive control. **f** Representative lung section from a COPD patient containing a lymphoid follicle stained with antibody against CD21 is shown. Representative lung sections from a **g**) control non-smoker and **h**) COPD patient stained with antibody against BAFF are shown.

Lymphoid follicles surrounding small airways defined as those with an airway perimeter between 2 to 4 mm in diameter were profiled in lung tissue samples from *N* = 29 male and *N* = 19 female smokers with severe and very severe COPD. Table 1 and Additional file 1: Figure S1 summarize the demographics of all subjects for the histologic assessment of lymphoid follicles by immunohistochemistry and the analysis flow for the exclusion criteria, respectively. Lymphoid follicles were identified as clusters of cells in H&E-stained sections and confirmed by immunohistochemical staining for CD79α on B-cells in patients with COPD (Fig. 6a-b). Females had significantly greater % airways positive for lymphoid follicles in airway perimeter 2-4 mm compared to those in males with COPD GOLD 3&4 (Fig. 6c). To evaluate the presence of follicular dendritic cells in lymphoid follicles, human tonsils were used as positive controls for CD21 (Fig. 3d-e). Representative images showed CD21+ staining in the center of a lymphoid follicle of COPD patients (Fig. 3f). Representative images of human lung tissues showed an increase in BAFF+ cells in the parenchyma of COPD compared to control non-smoker (Fig. 6g-h).

## Discussion

The most novel and important finding of our study was that female smokers have increased expression of lymphoid follicles in the small airways, which in a murine model was associated with increased expression of BAFF, foamy macrophages and oxidative stress markers. Interestingly, in ovariectomized female mice, these findings were absent, even in the presence of long-term cigarette exposure. Together, these data suggest that female sex hormones play a crucial role in the pathogenesis of airway-based lymphoid follicles. They may have important clinical implications as female smokers have increased risk of COPD and disease progression [19]. In mice with COPD-like pathologies, we have previously shown that smoke-exposed female mice display worse lung function and a greater burden of COPD changes in lungs including increased airway wall thickness, airway resistance and oxidative stress compared to those of male mice, and these changes were attenuated by ovariectomy [5, 14].

Previously, Hansen and colleagues showed that chronic smoke exposure increased the number of lymphoid aggregates in mice compared to air-exposed controls [11]. We extend these findings by demonstrating that female but not male or ovariectomized mice exposed to cigarette smoke for 6 months harbor lymphoid aggregates in the airways. Consistent with previous report [7], we showed evidence of B-cells and follicular dendritic cells infiltrating all lymphoid aggregates in female smoke-exposed mice. In human lungs, tertiary lymphoid follicles are almost always absent in the airways of healthy non-smoker. With smoking, however, a small number of these are observed in the walls of small airways. In COPD lungs, their numbers dramatically increase especially in patients with GOLD 3 and 4 disease [6]. These lymphoid follicles are composed of B cells, CD4+ T cells and follicular dendritic cells [6–8], with a majority of these follicles expressing Ki67+ proliferative cores [20]. However, our data revealed that only one of five representative COPD patients showed traces of Ki67+ cells without evidence of clear proliferative centers. Since our available samples are mostly COPD ex-smokers, differences in smoking history may explain this observed discrepancy, which requires further studies. A more recent study showed that some of these follicles stain positively for interleukin (IL21), a cytokine that promotes B cell proliferation in the germinal centers of lymphoid follicles [21], which are almost exclusively expressed in CD3+ T cells in patients with moderate and severe COPD compared to healthy controls [22].

The precise mechanism(s) by which lymphoid follicles are formed in patients with COPD is unclear. However, several murine studies have demonstrated the importance of B-cell activating factor (BAFF) [9], IL17A [10] and CXCL13 [23] in the formation of lymphoid follicles in mice after chronic smoke exposure. Intriguingly, in one study, BAFF+ macrophages were increased in parenchymal tissues of mice when exposed to cigarette smoke for 4 days [9]. Similarly, airway and parenchymal tissue-associated BAFF+ macrophages and lymphoid follicles were significantly increased in patients with COPD compared to control non-smokers [24]. An increase in *BAFF* gene expression has also been observed in lung tissues and in bronchoalveolar lavage fluid (BALF) of active smokers compared to non-smoking controls [9]. Belonging to the TNF family, BAFF is primarily expressed by macrophages and dendritic cells, and has been shown to be involved in proliferation, differentiation, and survival of B cells [25–27]. *BAFF* gene expression is consistently greater in CD3+, B220+ and CD11b + (macrophages) and CD11c + (dendritic) cells in female mice spleen compared to males, where CD11b + cells are the predominant source of BAFF [28]. Total *BAFF* gene expression has been shown to be reduced in estrogen receptor alpha knockout female compared to female wild type mice [28]. Consistent with these findings, treatment of mouse macrophage cell line (RAW264.7) with estradiol or interferon-alpha (INF-α) increased *BAFF* gene and protein expression [28]. Together, these data suggest that female sex hormone (estradiol) play a direct role in BAFF expression.

Oxidative stress has been shown to be an important factor in the contribution to COPD. In the context of COPD, Naz and colleagues identified oxidative stress-related metabolic shifts such as beta-oxidation, purine degradation and ratios of free carnitine to medium- and long-chain acylcarnitines in serum, which were significantly increased in women than in men with COPD [29]. BALF cell proteomics revealed significant upregulation of proteins involved in oxidative phosphorylation, which was differentially increased in women than men with COPD, thus supporting a role in the dysregulation of energy metabolism [30]. Multivariate modeling revealed a gender-specific phenotypic difference in the production of lipid mediators from cytochrome P450-derived epoxide products of linoleic acid and soluble epoxide hydrolase-derived products that were primarily upregulated in female smokers with COPD compared to healthy female smokers, and this effect was not observed in males [31]. Women also have consistently elevated circulating oxidative stress markers compared with men in both healthy non-smokers and smokers without COPD [32, 33]. Patients with severe COPD have increased iNOS and 3-nitrotyrosine expression in the whole lung tissues compared to healthy non-smokers [34]. Total 3-nitrotyrosine+ inflammatory cells (polymorphonuclear cells and macrophages) were significantly increased in induced sputum of patients with acute severe COPD exacerbation compared to patients with stable severe COPD [35]. In murine models of COPD, iNOS −/− mice were completely protected from chronic smoke-induced increase in 3-nitrotyrosine expression and emphysema compared to smoke-exposed wildtype mice [34].

Another important related observation was the increased accumulation of foamy macrophages in parenchymal tissues of mice after chronic smoke exposure compared to air-exposed mice [12, 36]. Consistent with these reports [12, 36], we showed a strong differential increase in foamy macrophages, *MMP12* and *CXCL2* gene expression in the parenchymal tissues of female mice compared to male mice. We also reported an increase in BAFF protein expression in mouse macrophage cell line in response to oxidative stress, suggesting a potentially relevant contribution to the formation of lymphoid aggregates. The precise driver of increased BAFF expression in lymphoid aggregates is not known. However, BAFF protein expression was previously reported to be up-regulated by hydrogen peroxide, TGFβ1 and IFNγ in murine macrophage cell line (RAW264.7) [37–39]. Consistent with this finding, we previously showed that female mice exposed to 6 months of cigarette smoke demonstrated a greater burden of oxidative stress than male or ovariectomized mice exposed to the same duration of cigarette smoke [5]. Thus, we speculate that female sex hormones (especially estradiol) may play a significant role in upregulating oxidative stress related to long-term cigarette exposure, which in turn may increase local expression of BAFF, leading to lymphoid aggregate formation. Future studies will be needed to validate this hypothesis.

Emphysema and the small airway disease are the major pathological changes in COPD. While lymphoid follicles are found in both small airways and parenchyma, earlier studies have shown that the presence of lymphoid follicles is closely associated with emphysema [40–43]. Since the small airway disease precedes emphysema [44] and destruction of alveolar attachments to the airway’s outer wall could link the airway inflammation driven by B cell to centrilobular emphysema [45], we speculate that the increased lymphoid follicles in the airways of female lungs with severe COPD might induce further destruction of alveolar attachments and emphysematous destruction around the airways. Although the mechanism by which lymphoid follicles and B cell-mediated inflammation are induced in COPD remains unclear, several papers have proposed potential factors including altered microbiome in the lung tissues [46] and autoimmunity [47] including production of anti-elastin, anti-tissue, and anti-endothelial antibodies. Given that females generally have greater susceptibility to auto-immune disease than males, the present finding that more airways with lymphoid follicles were found in females than males, suggests that auto-immune disorders might be involved in follicle formation. Consistent with this clinical observation, female mice harbored greater total number of lymphoid aggregates compared to males after chronic smoke exposure. Administration of antibodies against BAFF receptor-Fc blunted the formation of lymphoid aggregates and pulmonary antinuclear antibodies after chronic smoke exposure [9], suggesting an important auto-immune component. Further clinical studies are needed to evaluate auto-immune factors in male and female separately and identify a sub-population of COPD who may benefit from an immune-modulating therapy such as anti-BAFF antibody.

There were several important limitations to our study. First, chronic smoke exposure in mice with COPD-like pathologies (6 months) has been regarded to reflect mild COPD in humans, but the presence of lymphoid aggregates in mice suggest that it may be a reasonable model to study the biological implications associated with unique local environments in which these structures are formed. Secondly, it is important to note that the differential increase in *MMP12* and *CXCL2* gene expression arising from microdissected parenchymal tissues reflect the expression profile of a mixture of cells with potentially different roles and transcriptome. Although beyond the purview of this study, the use of single cell RNA sequencing technology may provide a more sensitive approach to pinpoint cell-specific molecular drivers responsible for the formation of lymphoid aggregates. Finally, our study did not evaluate the biological role of lymphoid follicles and their potential contributions to autoimmunity and respiratory infections; therefore, further studies are required to elucidate these mechanisms.

## Conclusions

In summary, female COPD lungs contain significantly more lymphoid follicles in the airways than in male COPD lungs, which may be related to female sex hormones and increased oxidative milieu, generated by long-term cigarette smoke exposure. Given the growing burden of COPD in female smokers and scarcity of disease-modifying treatment for this disease, there is a pressing need for additional studies to investigate their role in COPD pathogenesis especially in female patients.

## Supplementary information

**Additional file 1: Figure S1** Selection and exclusion criteria of subjects with for the assessment of airway-associated lymphoid follicles.

## Data Availability

All data generated or analysed during this study are included in this published article.

## Abbreviations

BAFF: B-cell activating factor
COPD: Chronic obstructive pulmonary disease
Cxcl2: Chemokine C-X-C motif ligand 2
Cyp1a1: Cytochrome P450 1a1
FFPE: Formalin-fixed paraffin embedded
GOLD: Global Initiative for Chronic Obstructive Lung Disease
H&E: Hematoxylin and eosin
Mmp12: Matrix metalloproteinase 12
Nqo1: NAD(P) H Quinone Dehydrogenase 1
OCT: Optimal cutting temperature
RAW 264.7: Mouse macrophage cell line
β2m: Beta-2 macroglobulin
