# Supplementary figures and images for: Sex differences in lymphoid follicles in COPD airways
Source: Respir Res. 2020 Feb 7;21:46. doi: 10.1186/s12931-020-1311-8 (PMC7006095; doi:10.1186/s12931-020-1311-8)

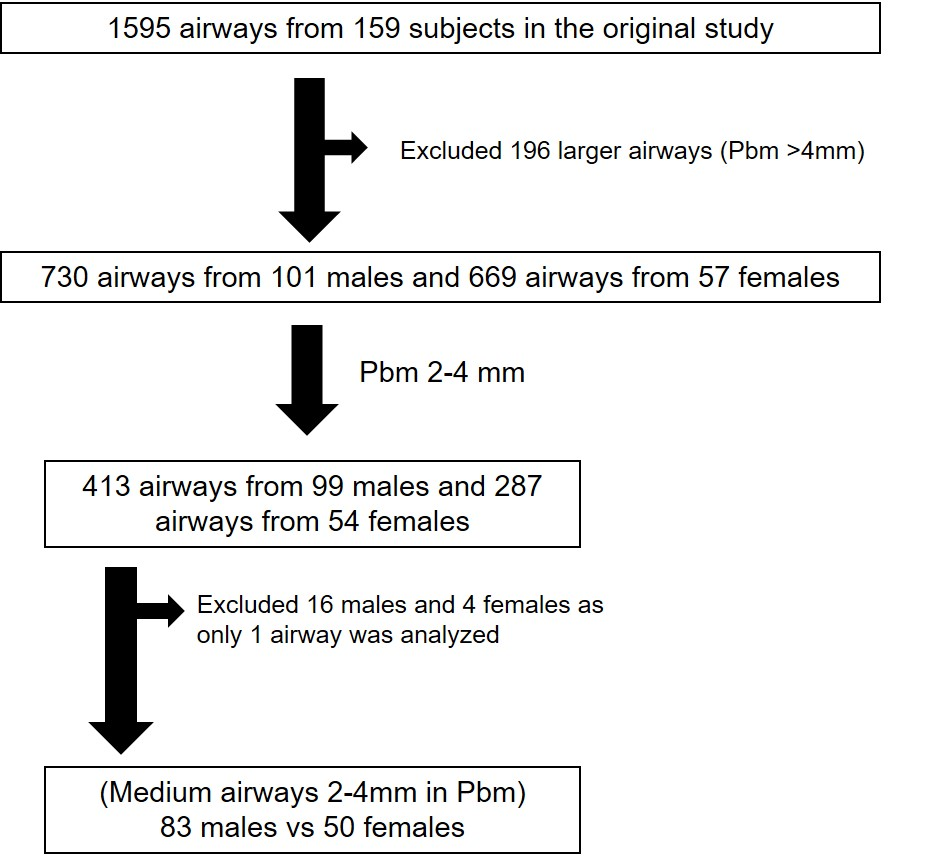

Supplement: Supplementary file 1 — Additional file 1: Figure S1 Selection and exclusion criteria of subjects with for the assessment of airway-associated lymphoid follicles. [file 12931_2020_1311_MOESM1_ESM.tif]
